# Effect of Different Silane Coupling Agents on the Bond Strength between Hydrogen Peroxide-Etched Epoxy-Based- Fiber-Reinforced Post and Composite Resin Core

**DOI:** 10.3390/dj11060142

**Published:** 2023-05-29

**Authors:** Sarah Adwani, Emad Elsubeihi, Ahmad Zebari, May Aljanahi, Keyvan Moharamzadeh, Haitham Elbishari

**Affiliations:** 1 College of Dentistry, Ajman University, Ajman P.O.Box 346, United Arab Emirates; 2 Dubai Dental Hospital, Dubai P.O. Box 505097, United Arab Emirates; 3 Hamdan Bin Mohammed College of Dental Medicine (HBMCDM), Mohammed Bin Rashid University of Medicine and Health Sciences (MBRU), Dubai P.O. Box 505055, United Arab Emirates

**Keywords:** fiber post, silane, dental adhesive, root canal treatment, bond strength

## Abstract

The aim of the study was to evaluate the effect of various silane coupling agents on the micro-push-out bond strength between a hydrogen peroxide-etched epoxy-based fiber-reinforced post and composite resin core. Seventy-five cross-linked epoxy-based fiber-reinforced posts were etched with 24% hydrogen peroxide for 10 min. Then they were divided into five groups according to various silane coupling agents and bonded to a composite core. A Universal Testing Machine was utilized to evaluate the push-out bond strength. In addition, all groups’ modes of failure were assessed. The push-out bond strength data in MPa were analyzed using ANOVA and a Tukey HSD post hoc test to reveal any difference between the groups. Results revealed that the application of a two-bottle silane coupling agent exhibited the highest bond strength, while the application of a one-bottle silane coupling agent demonstrated the lowest bond strength for a hydrogen peroxide-etched fiber post bonded to a composite core material, which was statistically significant (*p* < 0.05). The strongest association with the highest bond strength was found with the two-bottle silane coupling agent when compared to the one-bottle. The study highlighted that the application of a silane-coupling agent may affect the bond strength between composite and epoxy-based fiber-reinforced posts.

## 1. Introduction

Endodontically treated teeth commonly present with large amounts of missing tooth structure due to caries or existing restorations, contributing to high fracture rates [1,2]. This incidence can mainly be attributed to the loss of structural integrity associated with access cavity preparation, caries, and the inevitable flaring of the canal in the cervical area [3,4].

Thorough assessment of such teeth, including the efficacy of endodontic treatment, the quantity of dentine thickness, and post-endodontic direct restoration, which should be of high strength and acceptable clinical performance, all affect how long endodontically treated teeth will survive [3]. The clinician must take into consideration how challenging it is to provide core restoration to the remaining tooth structure, which depends on the remaining tooth structure, the core material, and the use of different adhesive cements.

There is increased interest in the use of resin composite material to restore endodontically treated teeth. This is attributed to the characteristics of the resin composite’s ability to bond to tooth structure as well as the advanced development of the physical properties of such materials. Despite the ability of resin composite materials to bond to remaining tooth structure, using fiber-reinforced posts is mandatory to retain the core in some endodontically treated teeth that have lost a significant amount of their structure. Fiber posts have an elastic modulus close to that of dentin, have adequate aesthetic properties when used under ceramic restorations, have the ability to bond to resin composite core material, have biocompatibility and resistance to corrosion, and require a shorter treatment time [5].

The retention and stability of the post system and core buildup are pivotal determinants for an effective and successful restoration. It has been reported that a coronal restoration improves the outcome of endodontically treated teeth [6], thus supporting the crucial nature of the bond between fiber posts and a resin composite core, as it enables the interface to transmit stresses under functional loading.

Factors influencing the retention of resin composite cores to the prefabricated post include post-surface treatment and post-head design, as well as the compositions of the post and resin composite core material [7].

To enhance the bonding of composite resin cores to posts, multiple surface treatments have been proposed. One of these treatments is the application of silane to the post surface. Silane is applied to the post surface before cementation and after roughening the post surface with sandblasting or the application of hydrogen peroxide. Numerous studies have evaluated the effects of silane agents on bond strength between resin composite cores and fiber-reinforced posts, including the effect of salinization on different matrix types of fiber-based posts [8], the influence of different surface treatments on the bond strength of silanted fiber posts to resin composite cores [9,10], and the impact of temperature on various silane coupling agents [11].

In a prior study, the bond strength of a composite resin to a glass fiber post treated with phosphoric acid, a silane coupling agent, and unfilled resin was assessed. The findings suggested that silane and unfilled resin applications could enhance the bond strength between glass fiber posts and resin composites [12].

The effect of silane coupling agents on the bond strength between a fiber-reinforced post and a composite resin core is controversial. Daneshkazemi et al. compared the effect of surface treatment on the bond strength of a composite core bonded to an epoxy-based fiber-reinforced post. The study reported that the application of the silane coupling agent had a significant effect on the bond strength of the glass fiber posts to composite resin when compared to the application of 30% hydrogen peroxide or 35% phosphoric acid to the fiber post [13]. On the other hand, Wrbas et al. showed that silane application did not increase the bond strength of a composite core to an epoxy-based fiber post [11].

The bond strength between glass fiber posts and composite resin cores was examined by Novais et al. in 2011 [14] in relation to the effects of air drying temperature and various silane coupling agents. Warm air drying following silane application did not result in an increase in the bond strength between the fiber-reinforced composite post and the composite core. However, when utilized with air drying at room temperature, the two-component silane produced stronger bond strength than all prehydrolyzed silanes [14].

The aim of this study is to evaluate the impact of various silane coupling agents on the bond strength between a hydrogen peroxide-surface-etched epoxy-based fiber post and a composite resin core.

The null hypothesis tested was that different types of silane coupling agents would have no effect on the bond strength between a hydrogen peroxide-surface-etched epoxy-based fiber post and a composite resin core.

## 2. Materials and Methods

### 2.1. Fabrication of the Experimental Models

We used 75 size 2 fiber-reinforced posts (Rely X fiber post, 3M ESPE, St. Paul, MN, USA). RelyX fiber posts have a tapered shape, with a diameter at the coronal end of 1.6 mm and 0.8 mm at the apical end. Moreover, these posts have no mechanical retentive features. The selected posts were etched using 24% H_2_O_2_ for 10 min. The posts were placed in a bur holder and then immersed in a container filled with 24% H_2_O_2_ for 10 min. Posts were then washed in an ultrasonic cleaner filled with deionized water for 10 min and then dried with oil-free air (an air-water syringe). The H_2_O_2_-etched fiber-reinforced posts were then randomly allocated to five different groups; each group consisted of 15 posts based on the type of silane used, then bonded to a resin composite core.

For control Group 1 (G1 = C), composite core material (Luxacore Z Dual, DMG) was applied to H_2_O_2_-treated Rely X fiber posts without silane coupling agent. For Group 2 (G2 = HV), a two-bottle silane coupling agent (Vitique; DMG) was applied to Rely X fiber posts. One drop of Vitique silane adhesive and one drop of Vitique silane activator were placed in a mixing palette (at a ratio of 1:1) and mixed with a micro-brush for 15 s. The mixed silane agent was applied to the surface of each post using a micro-brush and let set for 10 s. Then air was gently blown over the surface of the post. For Group 3 (G3 = H3M), a pre-hydrolyzed (one-bottle) silane coupling agent, RelyX Ceramic Primer (3M ESPE), was applied to Rely X fiber posts using a disposable micro-brush and let set for 60 s. Then air was gently blown over the surface of the post. For Group 4 (G4 = HMP), a pre-hydrolyzed (one-bottle) silane coupling agent, Monobond Plus (Ivoclar-Vivadent), was applied to Rely X fiber posts using a disposable micro-brush and let set for 60 s. Then air was gently blown over the surface of the post.

For Group 5 (G5 = HMN), a pre-hydrolyzed (one-bottle) silane coupling agent, Monobond N (Ivoclar-Vivadent), was applied to Rely X fiber posts using a disposable micro-brush and let set for 60 s. Then air was gently blown over the surface of the post.

**Figure 1:**
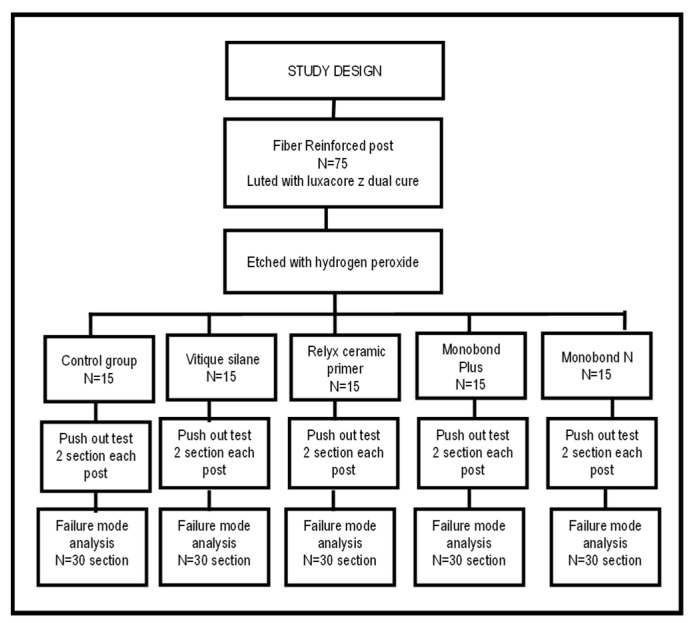
Study design showing the groups, number of posts in each group, and number of sections analyzed. N is the sample size.

To standardize the size of the composite core around each fiber post, a special type of conical transparent celluloid crown (TOR VM Ltd.) was used as a matrix for core buildup. Each matrix has a dimension of 10.0 mm height, 10.0 mm diameter at the cervical end, and 8.0 mm diameter at the coronal end. The study design is illustrated in Figure 1.

**Figure 2:**
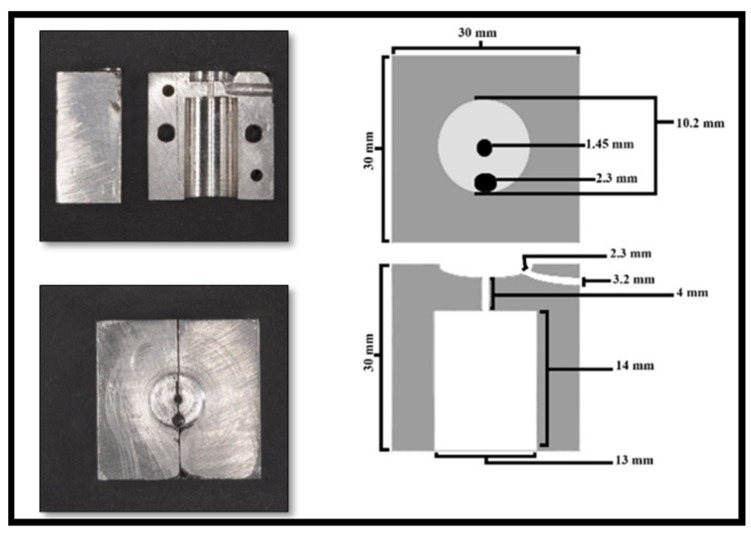
Customized mold used for core buildup.

**Figure 3:**
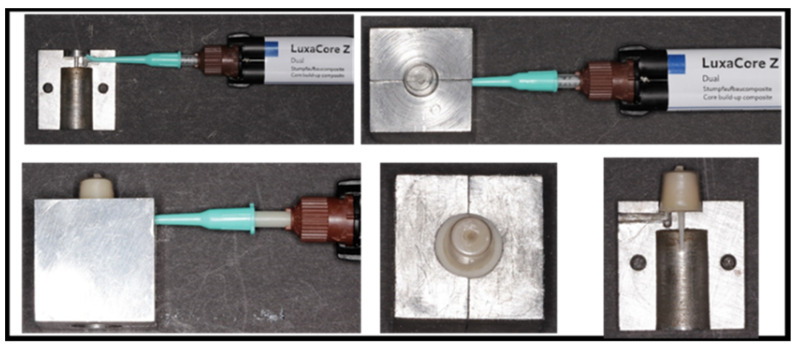
Assembly of mold components.

**Figure 4:**
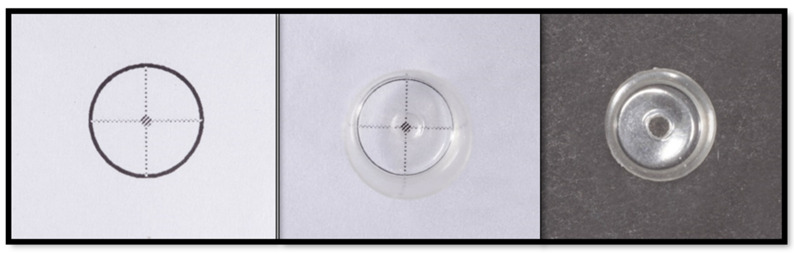
Matrix with a 1.6 mm hole to standardize post placement.

A custom-made metal mold was fabricated (Figure 2) to standardize the position of each fiber post within the composite core and the method of application of the composite core material. The mold consisted of two parts that were 15.0 mm wide, 30.0 mm long, and 30.0 mm high. When the two parts are assembled together, the post is held in the center of the celluloid crown form, which can sit over the upper part of the stainless-steel mold. Furthermore, a tunnel was made in the stainless-steel mold to allow the placement of the nozzle (Nanoceram-Bright, Nanohybrid light curing composite kit, DMP), which is used for injecting the composite core material around each post (Figure 3). To ascertain the centricity of each fiber post within the composite core material, a 1.6 mm hole was made in the coronal end of each celluloid crown form using a hot metal instrument (Figure 4). The position of each fiber post was, therefore, stabilized by the hole made in the customized stainless-steel jig at the cervical end and the hole made in the celluloid crown form at the coronal end. Each post protruded 2.0 mm through the hole made in the celluloid crown form following its placement in the stainless-steel base to ensure the stability of the fiber post’s position during the application of the composite core material. To standardize the position of the hole in the coronal end of each crown form, a template with the same diameter for the coronal end of the crown form was made with the aid of Adobe Photoshop CS6 computer software (Adobe Photoshop CS6, version 13.0 X 64, Macintosh version). In each template, a circle of 1.6 mm in diameter was drawn in the center, superimposed on the coronal end of the crown form, and a hole in the crown form was made using a hot instrument of 1.6 mm in diameter.

After silanization of the post surface, each post was positioned upright in the center of the custom metal mold and conical transparent celluloid crowns (TOR VM Ltd., Moscow, Russia). Then, the matrix was injected with composite resin using Luxacore Z Dual (DMG) until the excess protruded from the hole that had been made coronally. A sample was then cured using a light-emitting diode (LED) curing machine (LITEX 696 Cordless LED Curing Light, Dent America), in which each surface of the specimen was cured for 40 s.

**Figure 5:**
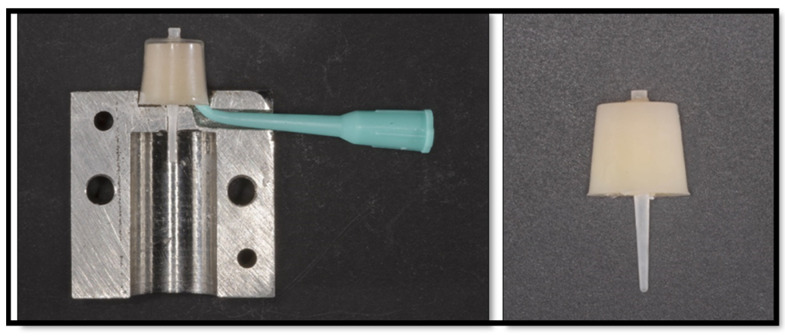
Post and core samples after light curing.

Then, the celluloid crown form was cut out with a number 15 scalpel blade and removed (Figure 5). Each composite core was further light cured for 20 s before being stored at 37 °C in 100% humidity in an incubator for 1 week.

### 2.2. Sectioning of the Samples

**Figure 6:**
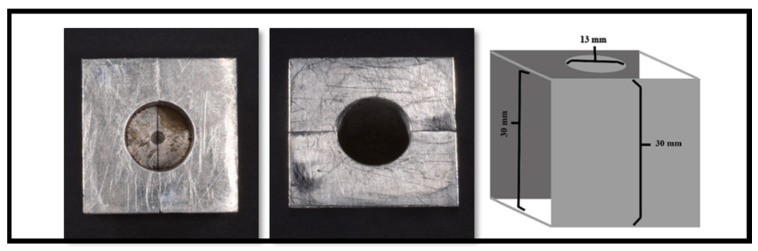
Customized mold for an acrylic base.

**Figure 7:**
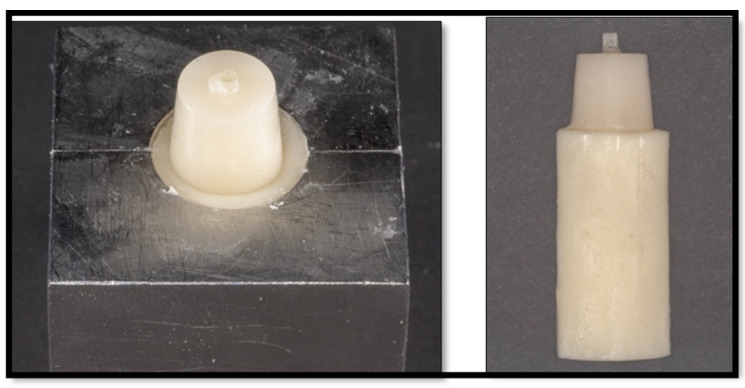
Sample immersed in the acrylic mold.

Another custom-made metal mold was designed with a specific dimension to hold the specimen (Figure 6). Vaseline was placed in the inner wall of the mold as a separating medium, and the mold was filled with self-cured acrylic resin (GC UNIFAST TRAD, GC International, Lucerne, Switzerland). The post was placed perpendicular to and at the center of the self-cured acrylic resin until the apical 1–2 mm of the core material was covered by the acrylic resin (Figure 7).

**Figure 8:**
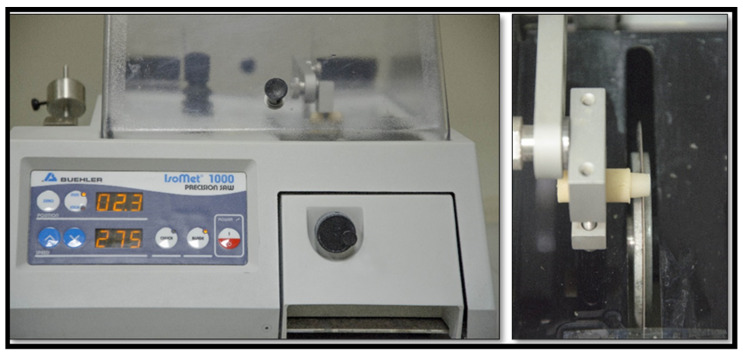
Cutting the sample into two slices with a thickness of 2 mm each.

Each specimen was placed in the saw machine (IsoMetTM 1000, 713-IPS-04427) perpendicular to the long axis of the saw disc, and the sample was cut into 2 mm sections (Figure 8) with a cutting blade thickness of 0.3 mm. The blade was calibrated to zero at the coronal end of the core to allow cutting at a distance of 0.7 mm with a speed of 275 rpm and a load of 100 g (Figure 8).

The thickness of each slice was measured using a digital caliper (Insize digital caliper, Standard Model). The coronal surface of each section was identified by placing a mark.

### 2.3. Measurement of the Lateral Surface Area of the Post

**Figure 9:**
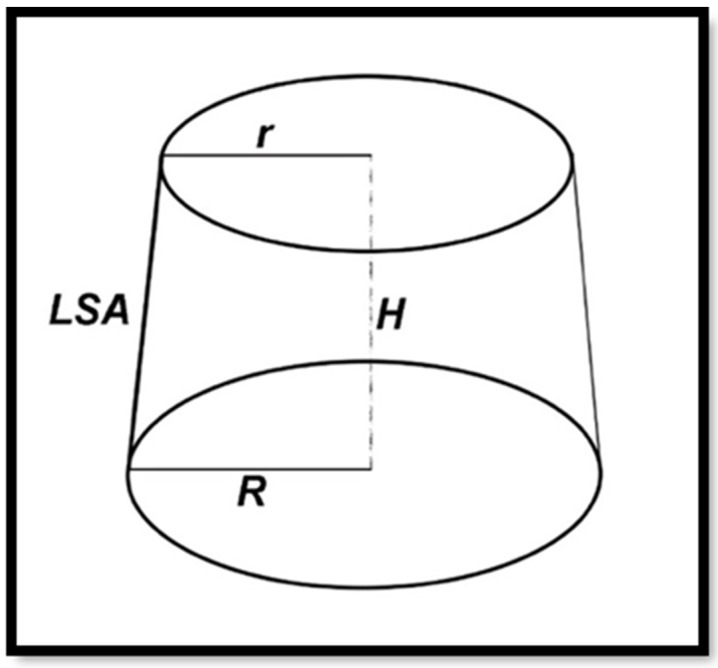
Schematic drawing showing the smallest and largest radius dimensions and the height of the post to be used for the calculation of the lateral surface area of the post (LSA = lateral surface area, H = thickness, R = largest radius, and r = smallest radius).

A stereomicroscope was used to take photographs of both the coronal and apical sides of each section in order to calculate the lateral surface area of each section using the following formula:

LSA = π (R + r)[(h2 + (R − r))2]0.5

where LSA is the lateral surface area, R is the largest radius, r is the smallest radius, and h is the slice thickness (Figure 9).

The method of measuring the lateral surface area of the post evolved from a previously published in vitro study [15], where it was comprehensively defined and outlined.

### 2.4. Measurement of Push-Out Bond Strength

**Figure 10:**
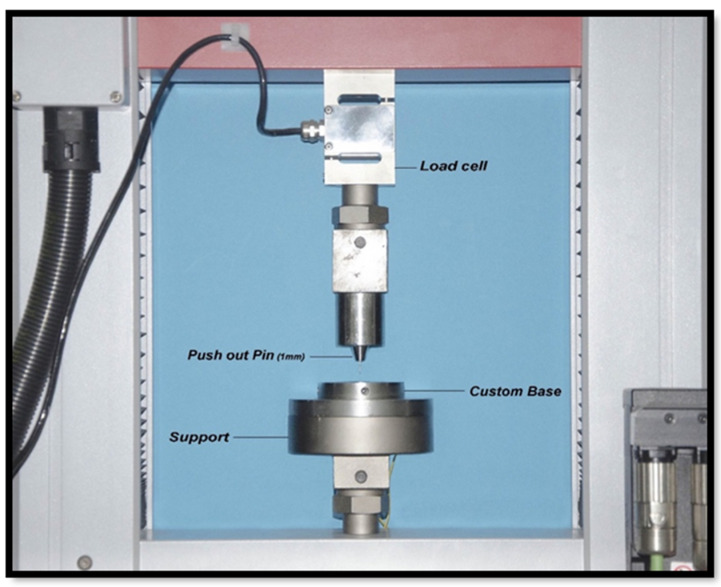
Universal testing machine components.

**Figure 11:**
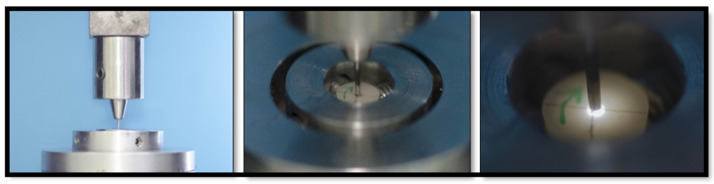
A custom stainless-steel base to fix the slice with LED light under the sample.

A custom-made stainless-steel base was used to hold the slices. The slice was placed with the apical side facing upwards under the Universal Testing Machine (Universal Testing Machine M350-5CT, Testometric, UK) (Figure 10) and an LED light under the hole of the base (Figure 11). Then, the base and slice were positioned within the Universal Testing Machine.

A stainless-steel rod with a 1 mm rounded pin at its end is attached to the loading cell and positioned in the center of the specimen. A continuous load at a cross-head speed of 0.5 mm/min until bond failure was determined and the value was recorded in newtons (N). Bond strength was calculated in megapascals (MPa). This was determined by dividing the force (N) by the lateral surface area (mm^2^) of each section.

### 2.5. Failure Mode Analysis

The analysis methodology evolved from a previously published research study [15], whereby it was comprehensively defined and outlined. Accordingly, the failure mode was checked under a stereomicroscope. A classification of the types of failure modes is illustrated in Table 1.

### 2.6. Statistical Analysis

The push-out bond strength data in MPa were analyzed using one-way analysis of variance (ANOVA). A Tukey HSD post hoc test was used to reveal any differences between the groups. Failure mode analysis was analyzed using the Chi-square test. The intra-examiner agreement for mode of failure was assessed using intra-class correlation statistics. Statistical analysis was carried out using SPSS software (SPSS version 21, 64-bit edition, IBM).

## 3. Results

**Figure 12:**
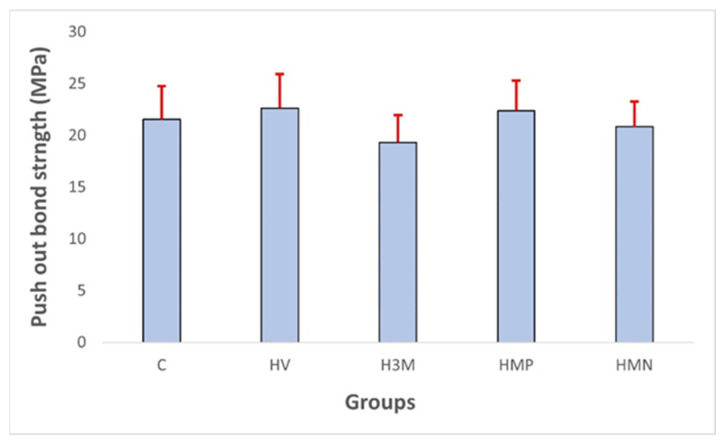
Push-out bond strength (in MPa) of the groups tested.

The mean and standard deviations of the push-out bond strength (in MPa) of all tested groups are summarized in Table 2. Among all groups, the HV group exhibited the highest bond strength (22.64 ± 3.27 MPa), while the H3M group resulted in the lowest bond strength (19.33 ± 2.63 MPa). The values of bond strength for other groups were 21.545 ± 3.202 MPa for the C group, 22.39 ± 2.89 MPa for the HMP group, and 20.83 ± 2.42 MPa for the HMN group. The values of push-out bond strength for all groups are illustrated in Figure 12. ANOVA statistical analysis revealed a highly significant difference between the groups (*p* < 0.05). The Tukey HSD statistical test revealed that the H3M group showed the lowest bond strength, which was a significant difference (*p* < 0.001) from the other groups. On the other hand, the difference between the other groups, namely the HMP, HMN, C, and HV groups, was not statistically different (*p* > 0.05) (Table 3).

**Figure 13:**
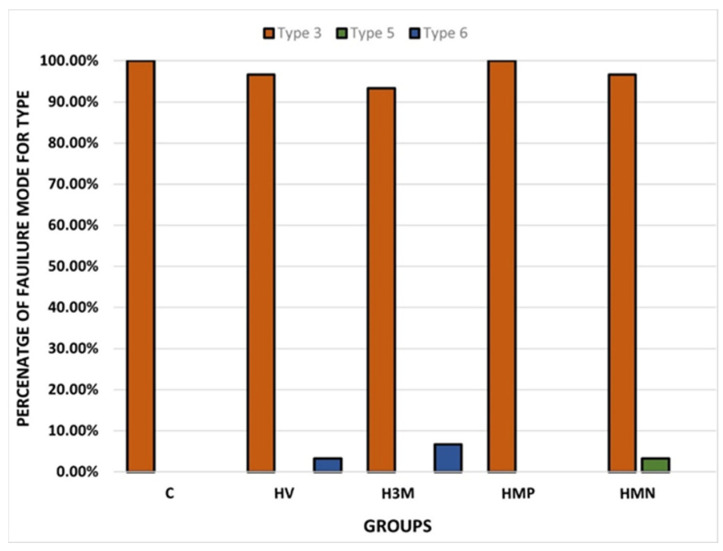
Percentage of types of failure modes within the groups.

No group has demonstrated cohesive failure within the post (type 1 failure mode) or cohesive failure within the composite core material (type 2 failure mode). Moreover, there has been no group that had mixed minimal adhesive failure (type 4 failure mode). On the other hand, the results revealed that the predominant mode of failure was total adhesive failure with no composite core material attached to the post surface (type 3 failure mode), which accounted for 97.3% of the total sample tested. Counts and percentages of different modes of failure for the different groups are presented in Table 4, and the percentage of each type of failure mode observed within the group is illustrated in Figure 13.

**Figure 14:**
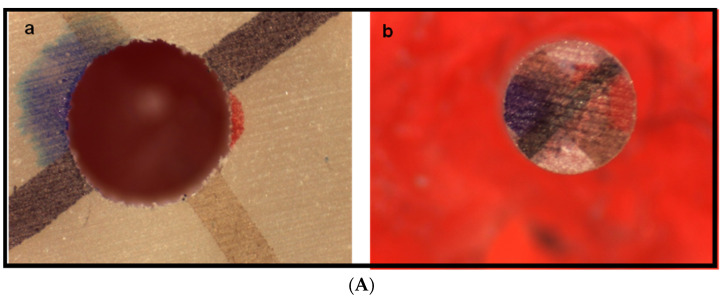

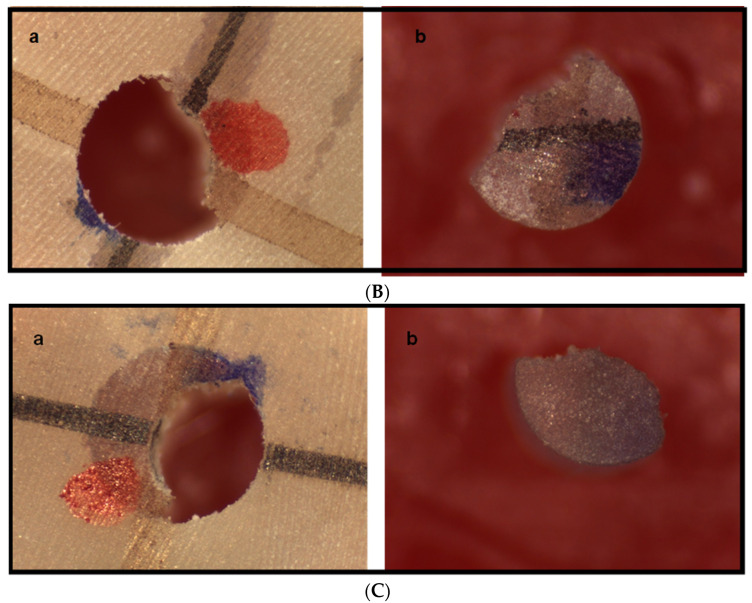
(**A**) Adhesive failure (type 3) (a: core, b: post). (**B**) Predominate adhesive failure (type 6) (a: core, b: post). (**C**) Moderate adhesive failure (type 5) (a: core, b: post).

The highest failure mode observed when a composite core was bonded to a Rely X fiber post without silane agents (Group 1) was total adhesive failure (type 3; 100%) (Figure 14A). No other mode of failure (including types 1, 2, 4, 5, and 6) was observed in Group 1.

For Group 2, in which a Rely X fiber post was bonded to a composite core material with the two-bottle silane coupling agent, Vitique silane, the highest failure mode observed was type 3 total adhesive failure (96.7%). This was followed by type 6, in which mixed failure with predominant adhesive failure occurred (type 6; 3.3%).

For Group 3, in which a Rely X fiber post was bonded to a composite core material with the one-bottle silane coupling agent, RelyX Ceramic Primer, the highest failure mode observed was type 3 total adhesive failure (93.3%). This was followed by type 6 (Figure 14B), in which mixed failure with predominant adhesive failure occurred (type 6; 6.7%).

For Group 4, in which a Rely X fiber post was bonded to a composite core material with the one-bottle silane coupling agent, Monobond Plus, the only failure mode observed was type 3 total adhesive failure (100%).

For Group 5, in which a Rely X fiber post was bonded to a composite core material with the one-bottle silane coupling agent, Monobond N, the highest mode of failure identified was type 3, in which total adhesive failure between the fiber post and composite core occurred (type 3; 96.7%). This was followed by type 5, in which mixed failure with moderate adhesive failure occurred (type 5; 3.3%).

Following analysis of the modes of failure in all groups, the predominant failure mode in all groups (groups 1, 2, 3, 4, and 5) was total adhesive failure (type 3), which ranged from 96.7% in Groups 2 and 5 to 93.3% in Group 3 and 100% in Groups 1 and 4 (Table 4).

In the analysis of the mode of failure of Group 2, in which a Rely X fiber post was bonded to a composite core material with the two-bottle silane coupling agent, Vitique silane, 96.7% of samples demonstrated type 3 total adhesive failure mode, while 3.3% of samples demonstrated type 6, in which mixed failure with predominate adhesive failure occurred (type 6; 3.3%).

In the analysis of the mode of failure of Group 4, in which a Rely X fiber post was bonded to a composite core material with the one-bottle silane coupling agent, Monobond Plus, 96.7% of samples demonstrated type 3 total adhesive failure mode, while 3.3% of samples demonstrated type 5 (Figure 14C), in which mixed failure with moderate adhesive failure occurred (type 5; 3.3%).

Intraclass correlation statistics were adopted to determine the intra-examiner agreement on failure mode assessments. The data was based on the measurement of 30 samples (6 samples from each group) twice on two separate occasions, two weeks later. The findings revealed a high level of intra-examiner agreement for the mode of failure (95%).

## 4. Discussion

The clinical outcome of a fiber-reinforced post and core restoration is determined by the materials used and the condition of the interfaces between the various materials [13,16].

Several surface treatments of fiber-reinforced posts have been investigated to maximize bonding of composite resin core material to fiber-reinforced posts with intermediate adhesive agents using either silane and/or adhesive application [17].

The coupling of fiber posts with epoxy resin to composite resin cores is hindered by the lack of chemical union between epoxy resins and methacrylate-based resins. [16].

In this investigation, a low-viscosity, highly-filled composite resin core material was bonded to a H_2_O_2_-etched fiber-reinforced post (Rely X post 3M ESPE) to evaluate the effect of different types of silane coupling agents on bond strength between the core and the post. The fiber-reinforced post used is cross-linked prefabricated epoxy resin mixed with zirconia fillers and reinforced with glass fibers. The bond strength between the methacrylate-based resin composites and the epoxy resin matrix of fiber-reinforced composites is relatively low when compared to dentin or enamel because of the lack of chemical union. This is caused by the difference in composition [16].

The core material used is specifically made for core buildup and was found to provide higher bond strength to fiber posts with an epoxy resin-based matrix as compared to other core materials [18,19]. Silane coupling agents are generally used to optimize the adhesion between inorganic surfaces and polymeric molecules. This is based on the ability of silane to increase the wettability of the fiber post’s surface and the creation of a chemical bond with an OH^−^ substrate in the fiber post, such as glass [20,21,22]. Yet, earlier research has shown that silane does not react effectively with the epoxy matrix [16,21]. Several chemical and mechanical surface treatment procedures for fiber-reinforced posts have been investigated to improve the bond strength between silane and the epoxy resin matrix. These pretreatments of the fiber-reinforced post surface, including sandblasting or chemical etching with hydrogen peroxide (H_2_O_2_), phosphoric acid, or hydrofluoric acid, have been investigated with the aim of exposing more glass fibers for improving retention of composite cores or luting cements to fiber-reinforced posts [16,20,23,24]. H_2_O_2_ is recognized as one of the surface treatments that may successfully dissolve the epoxy resin matrix, exposing the fibers and allowing them to be silanated [16,17,24].

In the current study, the application of the two-bottle silane coupling agent (Vitique, DMG) and the other one-bottle silane coupling agent exhibited a higher bond strength of the composite core to the hydrogen peroxide-etched fiber post as compared to the application of the Rely X ceramic primer, which exhibited the lowest bond strength. This result is in contrast with Novais et al.’s earlier findings [14], who showed that the two-bottle silane produced higher bond strength than all prehydrolyzed one-bottle silane coupling agents when it was used with air drying at room temperature (23 °C). The only similarity between the current results and Novais et al.’s study [14] was that the Rely X ceramic primer exhibited very low bond strength. This could be due to the difficulty of evaporating ethyl alcohol and water, which are solvents of the Rely-X ceramic primer (3M ESPE) and thus dissolve the bond [25].

All the silanes used in the current study have the same solvent composition (ethanol). It is assumed that ethanol has no effect on the bond strength between a fiber-reinforced post and a composite resin core. This assumption was proven by Kasraei et al.’s (2008) previous study [26].

The rate of silane hydrolysis could be affected by several factors, which may influence the nature of bonding between silane and the inorganic substrate. These include silane’s molecular structure, pH, temperature, solvent system, and humidity [27]. Furthermore, the rate of hydrolysis is affected by the solvent’s hydrophilicity. The hydrolysis rate reduces as the hydrophilicity of methanol, ethanol, and propanol decreases. This is due to the ability to separate “free” water molecules from bulk water (hydrogen-bonded network structure). After that, the “free” water molecule takes part in the silane hydrolysis reaction.

To explain the bonding mechanism of silane coupling in adhesive dentistry, various theories have been investigated [20]. According to the most historic chemical bonding theory, the reaction of the organo-functional group (R) and the hydrolyzed alkoxy groups (R’O3) with the resin matrix and the mineral substrate (glass or silica) of the composite material results in the formation of covalent bonds [28]. The more recent theory, known as the reversible hydrolytic bond mechanism theory, states that in the presence of water, the bonds between silane and mineral substrate are reversibly broken and recreated, allowing for stress release without loss of adhesion [28]. The chemical link is only achievable between the resin of the core material and the exposed fibers of the post because the silane agent can only chemically bridge resins and OH-covered inorganic substrates at the fiber post-composite core contact [20]. The strongly cross-linked polymers of the matrix in fiber posts, on the other hand, lack any functional group available for reaction, so the chemical reaction can occur only between the composite resin and the exposed glass fibers of the post [20]. The chemical or mechanical removal of the outermost layer of epoxy resin may leave more exposed fibers to react with the silane molecules. Previous research by De Sousa Menezes et al. (2011) [29] demonstrated that hydrogen peroxide may dissolve epoxy resin without harming glass fibers.

For dental applications, silane coupling agents are provided as either one- or two-bottle agents. The one-bottle variety contains a clear solution of pre-hydrolyzed (pre-activated) silane primers that contain solvents [30]. As a result of rapid solvent evaporation, such solutions may turn milky or hazy over time after repeated opening of the bottle due to the excess formation of siloxane oligomers or polymers, which are inactive components, making the silane coupling agent useless [30]. Consequently, the two-bottle system was introduced to prolong the shelf life and increase the reactivity [27]. In the two-bottle system, the first bottle contains unhydrolyzed silane monomer dissolved in ethanol, whereas the second bottle contains aqueous acetic acid. An equal amount of each bottle is mixed before immediate use to allow silane to hydrolyze and form the silanol (−SiOH) group, which then condenses, forming siloxane bonds [27].

In the current study, only one investigator assessed the mode of failure evaluations twice within two-week intervals. This assessment method was adopted from a previously published study [15]. The intra-examiner agreement of failure mode assessment was found to be 0.95. Earlier research suggested that a single operator performed failure mode analysis [29,30], but the coefficient of variation of measurements was only given in Alnaqbi et al. (2018) [15].

The predominant failure mode in all groups investigated in the current study was total adhesive failure (type 3 failure mode). This finding is in agreement with previous studies [11,28].

## 5. Conclusions

Within the limitations of this study, it can be concluded that the application of a silane coupling agent, pre-hydrolyzed or non-hydrolyzed, did not significantly affect the bond strength between the composite core and hydrogen peroxide-etched epoxy-based fiber-reinforced posts.

## 6. Clinical Implications

The choice of silane coupling agents is critical for improving the bond strength of composite cores to fiber-reinforced posts.

Clinicians should be aware of the composition of posts, surface treatments of posts, silane coupling agents, and composite core materials while selecting a combination of composite core-post materials to endodontically treat teeth.

## Figures and Tables

**Table 1:** Types of failure modes.

Types of Failure	Description
Type 1	Cohesive failure within the post
Type 2	Cohesive failure within the core
Type 3	Total adhesive failure (no core attached to post)
Type 4	Minimal adhesive failure (core material cover 75% of post surface)
Type 5	Moderate adhesive failure (core material cover 50% of post surface)
Type 6	Predominate adhesive failure (core material cover 25% of post surface)

**Table 2:** Descriptive statistics of push-out bond strength (in MPa) of all groups tested.

Post Number	Section Number	Rely X Post with Hydrogen Peroxide G1 = C	Rely X Post with Vitique Silane G2 = HV	Rely X Post with Relyx Ceramic Primer G3 = H3M	Rely X Post with Monobond Plus G4 = HMP	Rely X Post with Monobond N G5 = HMN
1	1 2	18.591 17.571	19.856 20.219	22.283 16.8	22.566 24.48	23.084 22.99
2	1 2	17.231 15.378	20.893 20.359	15.899 18.695	18.67 22.866	20.35 22.37
3	1 2	18.84 21.256	18.604 25.898	19.637 19.28	18.221 25.542	18.52 23.08
4	1 2	19.246 27.61	22.098 19.598	17.307 18.677	21.664 27.82	25.25 18.53
5	1 2	22.766 18.703	18.757 25.393	22.37 20.67	21.017 18.124	19.472 22.81
6	1 2	19.017 20.813	21.435 27.579	24.84 21.07	20.375 24.373	16.25 20.701
7	1 2	20.646 23.422	21.552 20.42	17.206 20.307	19.172 22.687	22.02 22.252
8	1 2	24.585 20.264	21.768 22.255	15.549 21.274	22.36 20.04	19.079 18.272
9	1 2	20.813 21.32	18.846 20.677	20.382 16.989	26.229 20.417	21.459 21.42
10	1 2	22.004 24.707	17.407 22.73	17.27 15.459	18.417 24.737	25.251 18.36
11	1 2	20.478 25.166	20.525 25.063	16.107 19.976	17.08 23.797	17.69 20.37
12	1 2	25.79 24.903	28.117 24.87	24.45 17.7	26.726 23.983	19.655 16.35
13	1 2	25.894 25.6	23.06 21.58	15.65 22.79	19.438 23.252	22.186 20.48
14	1 2	23.051 16.605	22.995 19.067	19.51 22.06	25.16 25.9	19.98 20.401
15	1 2	25.066 19.015	29.883 30.6	19.13 20.75	23.675 23.169	20.679 25.452
N = 15 (Total number of posts in each group)	N = 30 (Total number of sections)	M = 21.545	M = 22.64	M = 19.33	M = 22.39	M = 20.83
		SD = 3.202	SD = 3.27	SD = 2.63	SD = 2.89	SD = 2.42

N indicates sample size; M indicates mean (in MPa); SD indicates standard deviation.

**Table 3:** Post hoc Tukey HSD test of push-out bond strength.

Multiple Comparisons						
Dependent Variable: Bond Strength Tukey HSD						
	(J) Silane	Mean Difference (I–J)	Std. Error	Sig.	95% Confidence Interval	
					Lower Bound	Upper Bound
C	HV	−1.10090	0.74957	0.584	−3.1715	0.9697
	H3M	2.20880 *	0.74957	0.030	0.1382	4.2794
	HMP	−0.85353	0.74957	0.786	−2.9242	1.2171
	HMN	0.71960	0.74957	0.872	−1.3510	2.7902
HV	C	1.10090	0.74957	0.584	−0.9697	3.1715
	H3M	3.30970 *	0.74957	0.000	1.2391	5.3803
	HMP	0.24737	0.74957	0.997	−1.8233	2.3180
	HMN	1.82050	0.74957	0.114	−0.2501	3.8911
H3M	C	−2.20880 *	0.74957	0.030	−4.2794	−0.1382
	HV	−3.30970 *	0.74957	0.000	−5.3803	−1.2391
	HMP	−3.06233 *	0.74957	0.001	−5.1330	−0.9917
	HMN	−1.48920	0.74957	0.278	−3.5598	0.5814
HMP	C	0.85353	0.74957	0.786	−1.2171	2.9242
	HV	−0.24737	0.74957	0.997	−2.3180	1.8233
	H3M	3.06233 *	0.74957	0.001	0.9917	5.1330
	HMN	1.57313	0.74957	0.226	−0.4975	3.6438
HMN	C	−0.71960	0.74957	0.872	−2.7902	1.3510
	HV	−1.82050	0.74957	0.114	−3.8911	0.2501
	H3M	1.48920	0.74957	0.278	−0.5814	3.5598
	HMP	−1.57313	0.74957	0.226	−3.6438	0.4975

* The mean difference is significant at the 0.05 level.

**Table 4:** Counts and percentages of failure modes among groups tested.

Mode of Failure * Group: Cross-Tabulation								
			Groups					Total
			C	HV	H3M	HMP	HMN	
Mode of Failure	Type 1: Cohesive Failure within Post	Count %Within failure mode. %within groups	0 0.0% 0.0%	0 0.0% 0.0%	0 0.0% 0.0%	0 0.0% 0.0%	0 0.0% 0.0%	0 0.0% 0.0%
	Type 2: Cohesive Failure within Core	Count %Within failure mode. %within groups	0 0.0% 0.0%	0 0.0% 0.0%	0 0.0% 0.0%	0 0.0% 0.0%	0 0.0% 0.0%	0 0.0% 0.0%
	Type 3: Total Adhesive Failure	Count %Within failure mode. %within groups	30 20.5% 100.0%	29 19.9% 96.7%	28 19.2% 93.3%	30 20.5% 100.0%	29 19.9% 96.7%	146 100% 97.3%
	Type 4 (mixed): Minimal Adhesive Failure	Count %Within failure mode. %within groups	0 0.0% 0.0%	0 0.0% 0.0%	0 0.0% 0.0%	0 0.0% 0.0%	0 0.0% 0.0%	0 0.0% 0.0%
	Type 5 (mixed): Moderate Adhesive Failure	Count %Within failure mode. %within groups	0 0.0% 0.0%	0 0.0% 0.0%	0 0.0% 0.0%	0 0.0% 0.0%	1 100% 3.3%	1 100% 0.7%
	Type 6 (mixed): Predominant Adhesive Failure	Count %Within failure mode. %within groups	0 0.0% 0.0%	1 33.3% 3.3%	2 66.7% 6.7%	0 0.0% 0.0%	0 0.0% 0.0%	3 100.0% 2%
Total		Count %Within failure mode. %within groups	30 20.0% 100.0%	30 20.0% 100.0%	30 20.0% 100.0%	30 20.0% 100.0%	30 20.0% 100.0%	150 100.0% 100.0%

* C = Rely X Post with Hydrogen Peroxide, HV = Rely X Post with Vitique Silane, H3M = Rely X Post with Rely X Ceramic Primer, HMP = Rely X Post with Monobond Plus, HMN = Rely X Post with Monobond N.

## Data Availability

All data supporting reported results are available up on request.
